# Das geriatrische Syndrom Frailty im Kontext der Wiederaufnahme auf der Intensivstation: Studienprotokoll einer Mixed-Methods-Studie

**DOI:** 10.1007/s00063-025-01280-x

**Published:** 2025-04-23

**Authors:** Denise Schindele, Marc Moritz Berger, Irmela Gnass

**Affiliations:** 1 Paracelsus Medizinische Privatuniversität, Institut für Pflegewissenschaft und -praxis, Zentrum für Public Health und Versorgungsforschung, Strubergasse 21, 5020 Salzburg, Österreich; 2 Regionale Kliniken Holding Ludwigsburg, RKH Akademie, Kurt-Lindemann-Weg 10, 71706 Markgröningen, Deutschland; 3 RKH Klinikum Ludwigsburg, Klinik für Anästhesiologie, Intensivmedizin, Notfallmedizin und Schmerztherapie, Posilipostraße 4, 71640 Ludwigsburg, Deutschland

**Keywords:** Gebrechlichkeit, Geriatrische Patienten, Intensivversorgung, Risikofaktor, Wiederaufnahme, Frailty, Geriatric patients, Critical care, Risk factor, Readmission

## Abstract

**Hintergrund:**

Das Syndrom Frailty (dt. Gebrechlichkeit) ist gekennzeichnet durch den Rückgang physiologischer Reserven und eine verminderte Wiederstandsfähigkeit gegenüber internen und externen Stressfaktoren. Dies führt bei gebrechlichen Patienten neben einer erhöhten Mortalitätsrate zu einer erhöhten Anfälligkeit für Komplikationen während des Krankenhausaufenthalts, inklusive der Aufnahme auf eine Intensivstation (ITS). Der Einfluss von Gebrechlichkeit auf die Wiederaufnahme auf eine ITS ist in Deutschland unklar, da die bisherigen Erkenntnisse überwiegend aus nichtdeutschsprachigen Ländern stammen und nur begrenzt übertragbar sind.

**Ziel:**

Das primäre Ziel dieser Studie ist die Charakterisierung von Gebrechlichkeit (gemessen mit der Clinical Frailty Scale [CFS] ≥ 5) als potenzieller Risikofaktor für Wiederaufnahmen auf die ITS. Sekundär wird untersucht, welche Einflussfaktoren, insbesondere pflegerische und medizinische Maßnahmen auf Intermediate-Care- und peripheren Stationen, die Wiederaufnahme auf die ITS beeinflussen können.

**Methode:**

Die Studie folgt einem explanatorisch-sequenziellen Mixed-Methods-Design mit einer quantitativen und einer qualitativen Forschungsphase. In der quantitativen Phase wird eine prospektive Kohortenstudie durchgeführt, um den Zusammenhang zwischen Gebrechlichkeit und ITS-Wiederaufnahmen (≤ 30 Tage nach Entlassung oder während desselben Krankenhausaufenthalts) bei Patienten ≥ 65 Jahre zu untersuchen. Ergänzend wird in der qualitativen Phase eine Dokumentenanalyse durchgeführt, um Versorgungsmaßnahmen zu identifizieren, die einen Einfluss auf die Wiederaufnahme haben könnten.

**Erwartete Ergebnisse:**

Die primäre Hypothese lautet, dass Patienten mit Gebrechlichkeit (CFS ≥ 5) ein höheres Risiko für eine Wiederaufnahme auf die ITS haben als Patienten ohne Gebrechlichkeit. Diese soll in der quantitativen Phase überprüft werden. Im qualitativen Forschungsanteil erwarten wir, nichtintensivmedizinische Versorgungsmaßnahmen beschreiben zu können, welche auf die Wiederaufnahme Einfluss nehmen können.

**Zusatzmaterial online:**

Zusätzliche Informationen sind in der Online-Version dieses Artikels (10.1007/s00063-025-01280-x) enthalten.

## Abstract (en)

**Background:**

Frailty syndrome is characterized by a decline in physiological reserves and a reduced resistance to internal and external stressors. In addition to a higher mortality rate, patients who are frail are more prone to complications during hospitalization, including admission to an intensive care unit (ICU). Studies indicated that frailty syndrome may serve as a predictor of readmission to the ICU. The impact of frailty syndrome on readmission to an ICU in Germany remains to be elucidated, as extant research primarily originates from non-German-speaking countries and is only partially transferable.

**Aim:**

The primary objective of this study is to characterize the syndrome of frailty (measured using the Clinical Frailty Scale [CFS] ≥ 5) as a potential risk factor for readmission to the ICU. Secondly, the study will examine which factors, specifically nursing and medical measures on intermediate care and normal wards, can influence readmissions to the ICU.

**Method:**

The study employs an explanatory sequential mixed-methods design, comprising a quantitative and a qualitative research phase. In the quantitative phase, a prospective cohort study will be conducted to investigate the association between frailty syndrome and ICU readmissions (≤ 30 days after discharge or during the same hospital stay) in patients ≥ 65 years of age. Concurrently, a document analysis will be conducted in the qualitative phase to identify care measures that could influence readmission.

**Expected results:**

The primary hypothesis of this study is that patients diagnosed with frailty syndrome (CFS ≥ 5) exhibit a higher risk of readmission to the ICU compared to non-frail patients. This inquiry will be conducted in the quantitative phase of the study. The qualitative research component is designed to elucidate non-intensive care measures that may influence readmission.

## Hintergrund

Der Bedarf an intensivmedizinischer Versorgung steigt durch den demografischen Wandel international an [2, 36]. Gegenwärtig nähert sich das Durchschnittsalter kritisch kranker Patienten auf den Intensivstationen (ITS) dem Alter von 65 Jahren [10]. Der Anteil der kritisch kranken Patienten über 80 Jahre auf der ITS steigt schneller als jede andere Kohorte [10, 36]. Eine niederländische Beobachtungsstudie zeigt, einen 33 %-Anstieg von Patienten über 75 Jahre auf der ITS (2002–2005) [3]. Ähnliche Entwicklungen zeigen sich ebenfalls in weiteren europäischen Ländern [36] und Deutschland [22]. Intensivpatienten ≥ 80 Jahre machten von 2007 bis 2011 bereits 20 % aller Intensivpatienten in Deutschland aus [49].

Vor dem Hintergrund der demografischen Entwicklung auf den Intensivstationen rückt das geriatrische Syndrom Frailty (dt. Gebrechlichkeit) zunehmend in den Fokus der Behandlungsteams [6, 23]. Ältere, gebrechliche Patienten benötigen häufig nach einem chirurgischen Eingriff bzw. während eines Krankenhausaufenthalts aufgrund einer höheren Anfälligkeit für Komplikationen (z. B. Infektionen, Delir) eine intensivmedizinische Versorgung [2, 11, 36].

Gebrechlichkeit wird als mehrdimensionales, systemisches Syndrom definiert [8, 14]. Altersbedingte organische Veränderungen und Multimorbidität bedingen einen Rückgang der physiologischen Reserven [14, 22, 33, 44]. Gebrechlichkeit beeinträchtigt mehrere wichtige Körperfunktionen wie das endokrine System und die Gehirn‑, Muskel- und Immunfunktionen [13]. Gebrechlichkeit entsteht, wenn physiologische Vorgänge zur Aufrechterhaltung der Homöostase diesen Rückgang und weitere Beeinträchtigungen nicht mehr kompensieren können [14, 22, 33, 44]. Auf der ITS können die durch Gebrechlichkeit bedingten Einschränkungen in den Körperfunktionen die Behandlung akuter Erkrankungen wie z. B. Sepsis oder Organversagen erheblich erschweren, da sie die Kompensationsmechanismen des Körpers überfordern [13].

Die Erhebung von Gebrechlichkeit ist wie das Syndrom selbst komplex [37]. Das Alter per se steht nicht gleichbedeutend mit Gebrechlichkeit. Gebrechlichkeit muss daher vom chronologischen Alter abgegrenzt werden [46].

Zwischen 25 und 50 % der Intensivpatienten über 80 Jahre werden als gebrechlich eingestuft [22].

In der Literatur existieren unterschiedlichste Assessmentinstrumente [39]. Ein Scoping-Review von Church et al. [4] zeigt eine breite Anwendung der Clinical Frailty Scale (CFS) im klinischen Akutsetting. Ebenso zeigt die CFS zur Identifizierung von Gebrechlichkeit bei Intensivpatienten eine hohe prädiktive Validität (Odds Ratio [OR] zwischen 1,44 und 3,6) und Reliabilität für Mortalität und Krankenhausverweildauer [1, 4, 12, 15, 30, 35, 45]. Die Interrater-Reliabilität liegt insgesamt in einem hohen Bereich (Kappa zwischen 0,76 und 0,92), zeigt jedoch Abweichungen je nach Rater-Hintergrund [1, 12, 45]. Besonders gute Übereinstimmung wurde zwischen gleichprofessionellen Paarungen, wie Pflegenden untereinander (Kappa 0,92), beobachtet [12].

Die Verteilung von Ressourcen bzw. die Aufnahme von älteren Patienten und der Nutzen einer intensivmedizinischen Behandlung werden nicht erst seit der COVID-19-Pandemie diskutiert [18]. Eine erhöhte Wiederaufnahmerate von gebrechlichen Patienten steigert den Bedarf an den bereits begrenzten intensivmedizinischen Ressourcen [16, 24, 27, 29, 34, 48]. Personalmangel begrenzt zudem die Nutzbarkeit der verfügbaren Kapazitäten [25].

Fast jeder zehnte Intensivpatient wird während desselben Krankenhausaufenthalts erneut intensivmedizinisch behandelt [16]. Solche Wiederaufnahmen sind mit einer erhöhten Mortalitätsrate, längeren Krankenhausverweildauern und höheren Behandlungskosten verbunden [24, 27, 29, 48]. Nur wenige internationale Studien liefern wichtige Hinweise auf einen Zusammenhang zwischen Gebrechlichkeit und einer ITS-Wiederaufnahme [42, 43]. Es fehlen zudem Untersuchungen, die diesen Zusammenhang unter Berücksichtigung der deutschen Versorgungsstrukturen analysieren. Unterschiede in Gesundheitssystemen und Ressourcen erschweren die Übertragbarkeit internationaler Erkenntnisse und unterstreichen die Relevanz dieses Forschungsvorhabens.

## Forschungsfrage

Es ergeben sich folgende Forschungsfragen:

1. Ist Gebrechlichkeit (CFS ≥ 5) bei Patienten ≥ 65 Jahre, nach einer intensivmedizinischen Versorgung ein Risikofaktor für eine Wiederaufnahme auf die ITS (≤ 30 Tage nach Entlassung von der ITS und/oder während desselben Krankenhausaufenthalts)?
2. Welche Bedeutung hat die Umsetzung von Versorgungsmaßnahmen auf den Intermediate-Care-Stationen (IMC) und peripheren Stationen bei einer Wiederaufnahme auf die ITS (≤ 30 Tage nach Entlassung von der ITS und/oder während desselben Krankenhausaufenthalts) bei Patienten ≥ 65 Jahre mit Gebrechlichkeit (CFS ≥ 5)?

## Forschungsziel

Das primäre Forschungsziel der Studie liegt in der Charakterisierung von Gebrechlichkeit als möglicher Risikofaktor für eine Wiederaufnahme auf die ITS bei Patienten ≥ 65 Jahre. Als sekundäres Forschungsziel sollen mögliche Einflussfaktoren im Kontext der nichtintensivmedizinischen Versorgung auf die Wiederaufnahme identifiziert werden.

## Methodik

Dieses Studienprotokoll folgt den Empfehlungen für die Berichterstattung über Studienprotokolle für Observationsstudien und qualitative Studien (ObsQual-Checkliste [31]; Online-Zusatzmaterial).

### Studiendesign

Zur Bearbeitung der Forschungsfragen wird ein explanatorisch-sequenzielles Mixed-Methods-Design (MMD) verwendet. Das Studiendesign wird vom wissenschaftlichen Paradigma des Pragmatismus geleitet. Dieser erlaubt durch die Kombination von quantitativen und qualitativen Methoden, eine umfassende Beantwortung der Forschungsfragen und ein tieferes Verständnis im Themengebiet zu erlangen [5, 7, 19, 21, 28].

Die quantitative Phase wird der qualitativen Phase vorangestellt und die Ergebnisse der ersten Phase beeinflussen die Konzeption und Durchführung der zweiten Phase [5, 28].

**Abb. 1:**
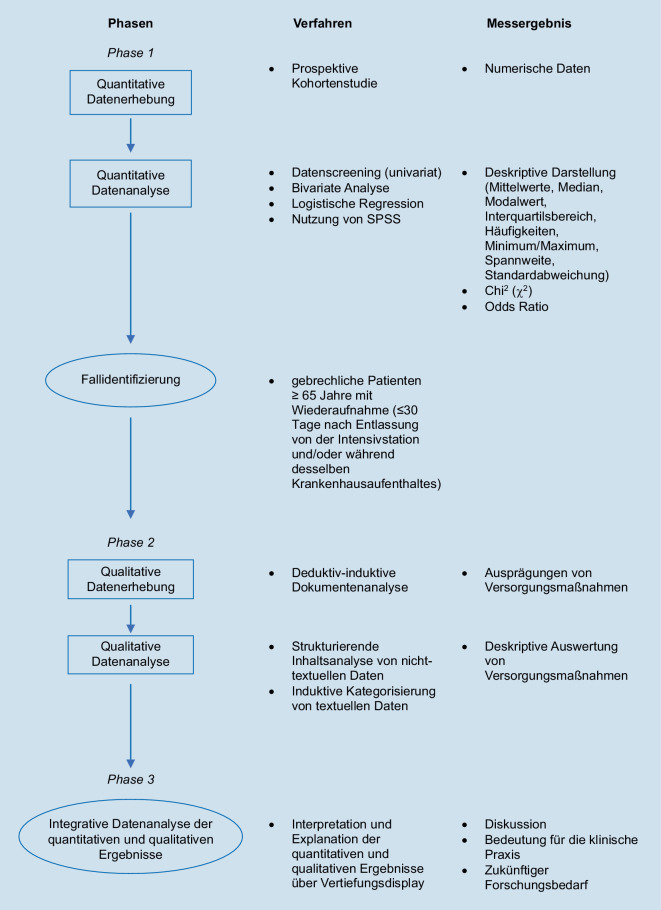
Prozesshafte Darstellung Forschungsverlauf

Der Verlauf des Forschungsdesigns ist in Abb. 1 prozesshaft dargestellt.

### Studiensetting

Die Studie wird auf einer interdisziplinären 20-Betten-Intensivstation einer Klinik der Maximalversorgung durchgeführt. Auf der ITS werden Patienten aller konservativen und chirurgischen Fachabteilungen (außer der herzchirurgischen Fachabteilung) behandelt. Auf der Station werden im Jahr ca. 1200 Patienten behandelt. Für die betrachtete ITS liegen bislang keine spezifischen Daten zur Wiederaufnahmerate vor. Auf der Station existieren bisher keine standardisierten Programme oder Routinen zur Identifizierung oder Behandlung von Gebrechlichkeit. In den standardisierten und routinemäßigen Assessmentinstrumenten, wie z. B. Erhebung eines Delirs, wird das Personal auf der ITS regelmäßig geschult.

Die Patientenaufnahmen umfassen sowohl elektive als auch Notfallaufnahmen. Geplante Aufnahmen erfolgen in der Regel im Rahmen elektiver chirurgischer Eingriffe, während ungeplante Aufnahmen durch akute Verschlechterungen des Gesundheitszustands notwendig werden. Im Gegensatz dazu unterscheiden sich ebenfalls die Entlassungen von der ITS: geplante Entlassungen sind proaktiv bis 15:00 Uhr an die Stationsprozesse angepasst, während ungeplante Entlassungen häufig auf akute Entscheidungen aufgrund dringend benötigter Bettenkapazitäten zurückzuführen sind.

Die Station verfügt über das elektronische Dokumentationssystem Metavision® (iMDSoft, Düsseldorf, Deutschland) und das Krankenhausinformationssystems ORBIS® (Dedalus HealthCare, Bonn, Deutschland).

### Studienpopulation

Die Auswahl erfolgt als Quotenstichprobe von September 2024 bis August 2025 und orientiert sich an der quantitativen Forschungsphase. Die angestrebte Größe der Stichprobe beträgt 55 Wiederaufnahmen. Studien zeigen eine durchschnittliche Wiederaufnahmerate von ca. 10 % [16]. Basierend auf diesen Daten kann davon ausgegangen werden, dass die benötigte Anzahl an Wiederaufnahmen auf der beschriebenen ITS innerhalb eines Studienzeitraums von 12 Monaten erreicht werden kann. Sollte die erforderliche Stichprobengröße nicht erreicht werden, ist in Absprache mit dem Studienleitungsteam und den zuständigen Gremien eine Verlängerung des Rekrutierungszeitraums vorgesehen.

Die Stichprobe für die qualitative Forschungsphase wird aus den Wiederaufnahmen der quantitativen Phase gezogen. Hierbei liegt der Fokus auf Patienten mit CFS ≥ 5, um tiefere Einblicke in die spezifischen Einflussfaktoren zu gewinnen.

Die Stichprobe für beide Forschungsanteile wird aus der gleichen Population gezogen. Die Stichprobe bilden Patienten ≥ 65 Jahre, welche auf der oben beschriebenen ITS behandelt werden. Es werden akut bzw. elektiv aufgenommene Patienten aller chirurgischen und konservativen Fachabteilungen (außer der herzchirurgischen Fachabteilung) eingeschlossen. Patienten, die der Verwendung der Daten nicht zustimmen und nicht die beschriebenen Merkmale aufweisen, werden ausgeschlossen. Der Beobachtungszeitraum erstreckt sich auf ≤ 30 Tage nach Entlassung von der ITS bzw. während des gesamten Krankenhausaufenthalts.

### Forschungsphasen

#### Phase 1: quantitative Phase

In der ersten Phase wird eine monozentrische, prospektive Kohortenstudie auf einer interdisziplinären Intensivstation durchgeführt. Die erste Phase hat das Ziel, die folgende Nullhypothese zu überprüfen:

Gebrechliche Patienten (CFS ≥ 5) haben nach einer intensivmedizinischen Behandlung das gleiche Risiko für eine Wiederaufnahme auf die ITS (≤ 30 Tage nach Entlassung von der ITS und/oder während desselben Krankenhausaufenthalts) wie nichtgebrechliche Patienten.

##### Quantitative Datenerhebung.

Die Patientendaten werden anhand des elektronischen Dokumentationssystems der Klinik bzw. Station erhoben. Die benötigten Daten und Variablen werden in Microsoft Excel (Version 2021) übertragen und im ersten Schritt pseudonymisiert. Die Datenextraktion erfolgt ausschließlich durch Mitglieder des Forschungsteams, das die relevanten Informationen aus Metavision® und ORBIS® manuell extrahiert. Daten, die nicht automatisch in der Dokumentation enthalten sind, werden anhand validierter Checklisten bzw. Assessmentinstrumente seitens der Mitglieder des Forschungsteams gesondert erhoben. Diese sind:

- Gebrechlichkeit anhand der CFS [40]
- Komorbiditäten anhand des Charlson-Komorbiditäts-Index (CCI; [38])
- Erhebung der Pflegebedürftigkeit anhand der Aktivitäten des täglichen Lebens (ADL) Score nach Katz [26]

Die Zielgrößen der quantitativen Datenerhebung finden sich in Tab. 1 und 2.

**Tab. 1:** Zielgrößen der unabhängigen Variable

	Variable	Definition	Cut-off	Erhebung/Quelle
Unabhängige Variable	Gebrechlichkeit	CFS ≥ 5, gebrechlich	CFS ≤ 4, nicht gebrechlich	Befragung Patient oder/und An- und Zugehörige, Pflegestatus in Patientenakte (durch Forschungsgruppe)
Kontrollvariablen	Alter	≥ 65 Jahre	< 65 Jahren	Patientenakte
	Geschlecht	Männlich, weiblich, divers	–	Patientenakte
	CCI	Gemessen bei Aufnahme ITS, Punkte 0 bis ≥ 5	–	Anamnese Patientenakte (durch Forschungsgruppe)
	ADL-Score	Gemessen bei Aufnahme ITS, Punkte 0–6	–	Befragung Patient oder/und An- und Zugehörige, Pflegestatus in Patientenakte (durch Forschungsgruppe)
	SOFA-Score	Gemessen bei Aufnahme ITS (spätestens 24 h nach Aufnahme), Punkte 0–24	–	Patientenakte (durch Stationsärzte, Routineerhebung)

*ADL* Aktivitäten des täglichen Lebens, *CCI* Charlson-Komorbiditäts-Index, *CFS* Clinical Frailty Scale, *ITS* Intensivstation, *SOFA* Sequential Organ Failure Assessment

**Tab. 2:** Zielgröße der abhängigen Variable

	Variable	Definition	Cut-off	Erhebung/Quelle
Abhängige Variable	Wiederaufnahme auf die ITS	≤ 30 Tage nach Entlassung von der ITS und/oder während desselben Krankenhausaufenthalts	> 30 Tage nach Entlassung von der ITS und/oder nach Krankenhausentlassung	Patientenakte
		Jede Aufnahmediagnose, welche zur Wiederaufnahme führt	–	Patientenakte
		Jeder Zeitpunkt der Wiederaufnahme, gemessen in Stunden der Versorgung außerhalb der ITS	–	Patientenakte
		Jede nachgelagerte Station innerhalb des Krankenhauses (IMC, bettenführende Station)	–	Patientenakte
Kontrollvariablen	Intensivaufenthalt	Länge des primären ITS-Aufenthalts, gemessen in Stunden	–	Patientenakte
	Zeitpunkt der ITS-Entlassung	Entlassung beim primären ITS-Aufenthalt, gemessen in Uhrzeit	–	Patientenakte
	Art der ITS-Entlassung	Geplant, ungeplant; definiert nach Stationsablauf und Verlegungsplan	Geplant ≤ 14:59 Uhr, ungeplant ≥ 15:00 Uhr	Patientenakte
	Intensivmedizinische Maßnahmen beim primären ITS-Aufenthalt	Invasive Beatmung mittels Endotrachealtubus oder Tracheostoma, gemessen in Stunden	–	Patientenakte
		Kontinuierliches Nierenersatzverfahren, gemessen in Stunden	–	Patientenakte
		Katecholamintherapie, gemessen in verabreichter Gesamtmenge in mg	–	Patientenakte
		Analgosedierung in verabreichter Gesamtmenge in mg	–	Patientenakte
	Komplikationen während des ITS-Aufenthalts	Ventilatorassoziierte Pneumonie, Auftreten während der Beatmungszeit Diagnose gesichert durch Labor, RöThx Auftreten einer Pneumonie ≥ 48 h nach endotrachealer Intubation mit Beatmung (Diagnose nach KISS-Kriterien)	Pneumonie vor dem 4. Beatmungstag Auftreten einer Pneumonie nach Extubation Keine gesicherte Diagnose der Pneumonie	Abruf über Hygienefachperson der Station
		Delir, Auftreten während des ITS-Aufenthalts, Diagnose ermittelt durch den ICDSC (gemessen alle 8 h oder bei Bewusstseinsveränderung)	–	Patientenakte, regelmäßige Erhebung durch Pflegende (Routineerhebung)

*ICDSC* Intensiv Care Delirium Checklist, *IMC* Intermediate-Care-Station, *ITS* Intensivstation, *KISS *Krankenhaus-Infektions-Surveillance-System, *RöThx *Röntgenaufnahme des Thorax

##### Stichprobengröße.

Die exakte Berechnung der Stichprobengröße gestaltet sich aufgrund der heterogenen Wiederaufnahmeraten in der Literatur (3–28 %; [9, 16, 24, 27, 29, 34, 42, 48]) als herausfordernd. Die Raten variieren zudem je nach strukturellen Bedingungen der jeweiligen Intensivstationen. Zur Orientierung wurden zwei relevante Studien herangezogen, die einen statistisch signifikanten Zusammenhang zwischen Gebrechlichkeit und Wiederaufnahme auf die Intensivstation untersucht haben. Sanchez et al. (2020; [43]) analysierten 977 Patienten und berichteten eine Wiederaufnahmerate von < 5 %. Ruiz de Gopegui Miguelena et al. (2021; [42]) untersuchten 90 Patienten ≥ 70 Jahre und fanden eine deutlich höhere Rate von 28 %. Da die Wiederaufnahmerate in der Studie von Sanchez et al. (2020; [43]) näher am Literaturdurchschnitt von 10 % [16] liegt, wurde diese als Referenzstudie für die Berechnung der Stichprobengröße verwendet.

Die Berechnung der Stichprobengröße orientiert sich an der Identifikation von Gebrechlichkeit als potenzieller Risikofaktor für eine Wiederaufnahme auf die Intensivstation. Aus den Daten von Sanchez et al. (2020; [43]) wurde zunächst die Odds Ratio (OR) berechnet (OR = 1,605), die ein erhöhtes Risiko für die Wiederaufnahme bei gebrechlichen Patienten bestätigt. Basierend darauf wurde ein Berechnungsansatz mit Fokus auf das primäre Outcome verfolgt. Hierfür wurde die errechnete OR in einen Pearson-Korrelationskoeffizienten (r = 0,404) umgerechnet. Eine statistische Power von 80 % sowie ein Signifikanzniveau von 5 % wurden festgelegt. Daraus resultierte eine erforderliche Stichprobengröße von *n* = 45 Wiederaufnahmen für ein statistisch signifikantes Ergebnis (G*Power 3.1). Um Drop-outs zu kompensieren, wird eine Stichprobengröße von *n* = 55 angestrebt. Eine genaue Aussage über die Gesamtpopulation (N) kann im Rahmen dieser Studie nicht getroffen werden. Die Berechnung orientiert sich an einer Fokuspopulation von Wiederaufnahmen auf die Intensivstation, unabhängig davon, ob Patienten als gebrechlich eingestuft wurden. Da die Erhebung des Frailty-Status im Rahmen der Studie erfolgt und bislang keine systematische Erfassung stattfindet, wäre eine Vorauswahl ausschließlich gebrechlicher Patienten methodisch nicht möglich. Stattdessen erfolgt eine nachträgliche Stratifizierung der Patientengruppe anhand der CFS (CFS ≥ 5). Dies stellt sicher, dass eine Analyse des Zusammenhangs zwischen Gebrechlichkeit und einer Wiederaufnahme unter realen klinischen Bedingungen erfolgen kann.

##### Quantitative Datenanalyse (statistische Methoden).

Die Daten der prospektiven Kohortenstudie werden zunächst deskriptiv ausgewertet und dargestellt. Im Rahmen der univariaten Analyse erfolgt, je nach Skalenniveau, die Darstellung der Ergebnisse als absolute und relative Häufigkeiten in %, Modalwert, Median, Minimum/Maximum, Interquartilsbereiche, Mittelwert, Spannweite und Standardabweichung.

Im Rahmen der bivariaten Analyse soll mittels des Verfahrens durch Chi^2^ (χ^2^) der Zusammenhang zwischen der unabhängigen Variable Gebrechlichkeit und den abhängigen Variablen (Wiederaufnahme, Delir und ventilatorassoziierte Pneumonie [VAP]) dargestellt werden.

Zur vertiefenden statistischen Analyse wird die logistische Regression eingesetzt, um den Einfluss von Gebrechlichkeit (CFS ≥ 5) auf ITS-Wiederaufnahmen zu untersuchen. In das Modell werden Kontrollvariablen wie z. B. Alter, Geschlecht und Länge des primären ITS-Aufenthalts einbezogen, um den potenziellen Einfluss von Störgrößen zu kontrollieren. Fehlende Daten werden durch multiple Imputation ergänzt, sofern der Anteil fehlender Werte ≤ 20 % beträgt. Sensitivitätsanalysen werden durchgeführt, um die Robustheit der Ergebnisse zu überprüfen.

Subgruppenanalysen werden durchgeführt, um Unterschiede in der Wiederaufnahmerate zwischen Geschlechtern, Altersgruppen und Gebrechlichkeitsgrad zu untersuchen. Wechselwirkungen zwischen Gebrechlichkeit und anderen Variablen wie Komorbiditäten werden durch Interaktionstests innerhalb der logistischen Regression geprüft.

Die primäre Analyse dient der Überprüfung der Nullhypothese. Sekundäre Analysen untersuchen den Einfluss von Gebrechlichkeit auf Delir und VAP. Zur Interpretation der Wahrscheinlichkeit einer Wiederaufnahme wird zudem die OR abgeleitet.

#### Phase 2: qualitative Phase

In der zweiten Phase der Studie wird eine Dokumentenanalyse der Patientenakten anhand der qualitativen Inhaltsanalyse nach Mayring (2022; [32]) vorgenommen. Die Analyse erfolgt deduktiv-induktiv: Der deduktive Ansatz basiert auf einem initialen Kategoriensystem, das durch Literaturrecherche entwickelt wird. Der induktive Ansatz ermöglicht es, während der Analyse neu auftretende Kategorien zu identifizieren und in das Kategoriensystem zu integrieren.

##### Qualitative Datenerhebung.

Die Daten umfassen ärztliche und pflegerische Dokumentationen, Versorgungsmaßnahmen und Verlaufseinträge aus der elektronischen Patientenakte der Klinik. Auf der Grundlage der Diagnose der Wiederaufnahme wird ein initiales Kategoriensystem erstellt, das pflegerisch-therapeutische Maßnahmen und medizinische Interventionen abdeckt. Dieses Kategoriensystem wird iterativ weiterentwickelt und durch induktiv gewonnene Erkenntnisse ergänzt, um ein umfassendes Bild der Versorgungsfaktoren zu erhalten [32].

##### Stichprobengröße.

Die Stichprobe der qualitativen Phase wird aus den Ergebnissen der prospektiven Kohortenstudie gezogen. Die qualitative Analyse konzentriert sich auf Patienten mit Gebrechlichkeit (CFS ≥ 5) und ITS-Wiederaufnahmen. Die Stichprobengröße wird flexibel anhand des Prinzips der theoretischen Sättigung bestimmt, wobei ca. 15–20 Fälle erwartet werden, um eine ausreichende inhaltliche Tiefe zu gewährleisten.

##### Qualitative Datenanalyse.

Die Datenanalyse im qualitativen Forschungsanteil orientiert sich am Vorgehen einer strukturierenden Inhaltsanalyse. Die Analyse von nichttextuellen Daten erfolgt anhand des Kategoriensystems und wird in Häufigkeitstabellen dargestellt und deskriptiv statistisch ausgewertet.

Die Analyse der textuell-schriftlichen Daten erfolgt in zwei Schritten. Neu erworbene Erkenntnisse werden kategorisiert und können somit operationalisiert werden. Diese neuen Kategorien werden iterativ in das Kategoriensystem integriert, wodurch eine umfassende und flexible Analyse möglich wird [32].

#### Phase 3: integrative Analyse im Mixed-Methods-Design

In diesem letzten Schritt des MMD werden die Datensätze aus der quantitativen und qualitativen Phase gemeinsam abgebildet, um vertiefende Erkenntnisse beschreiben und erklären zu können [5, 28].

Aus diesem Grunde eignet sich zur Darstellung und Analyse der beiden Datensätze ein Vertiefungsdisplay [28].

Ausgangspunkt für die integrative Datenanalyse sind die Ergebnisse aus dem quantitativen Forschungsteil. Das zu vertiefende Element aus dem quantitativen Forschungsanteil bildet die Wiederaufnahme auf die ITS bei gebrechlichen Patienten. Die qualitativen Daten können ggf. dazu beitragen, Hintergrundinformationen für bestimmte Trends, z. B. Häufigkeit einer bestimmten Aufnahmediagnose, zu liefern.

## Ethische Überlegungen

Die Genehmigung der Ethikkommission der Landesärztekammer Baden-Württemberg liegt vor (Aktenzeichen F‑2024-056). Zudem wurde die Studie beim Deutschen Register Klinischer Studien unter DRKS00034893 registriert.

Aufgrund des nichtexperimentellen Studiendesigns entsteht für die Patienten keine Gefährdung ihrer geistigen bzw. körperlichen Gesundheit. Da die Studie auf Daten zurückgreift, welche routinemäßig auf der Station bzw. welche durch den Einsatz von validierten nichtinvasiven Assessmentinstrumenten erhoben werden, entstehen für die Patienten keine zusätzlichen Belastungen oder Risiken. Die Behandlung der Patienten auf der Intensivstation bzw. nach der Entlassung von der ITS wird durch die Forschung nicht beeinträchtigt oder beeinflusst.

Die erhobenen Daten werden pseudonymisiert, indem sie mit einem eindeutigen Code versehen werden. Die Zuordnung der Codes zu den personenbezogenen Daten erfolgt getrennt und wird ausschließlich durch autorisiertes Personal verwaltet. Der Schutz dieser Daten wird durch die Einhaltung der Richtlinien der Datenschutzgrundverordnung (DSGVO) gewährleistet, sodass auch hier von keinem Risiko für die Patienten ausgegangen werden kann.

Die Teilnahme an der Studie ist freiwillig und die Einwilligung kann jederzeit vom Patienten selbst oder dem Betreuer widerrufen werden, ohne dass den Patienten dadurch Nachteile entstehen. Das Vorgehen bei der Aufklärung der Patienten orientiert sich an den internationalen Leitlinien zur guten klinischen Praxis [47]. Es ist davon auszugehen, dass der größte Anteil der Patienten zu Beginn der Datenerhebung, Aufnahme auf die ITS, zunächst nicht einwilligungsfähig sind. Die Einwilligungsfähigkeit wird zeitnah geprüft und eine Einwilligung wird sobald als möglich angestrebt. Die Aufklärung erfolgt schriftlich und persönlich durch eine Person der Forschungsgruppe. Die Aufklärung der Patienten selbst wird dabei ehestmöglich bei Entlassung bzw. nach Entlassung von der ITS vorgenommen. Bei Sprachbarrieren wird ein klinikinterner Dolmetscher hinzugezogen und die Aufklärung in entsprechender Sprache, mittels Übersetzungsprogrammen, übersetzt.

## Diskussion

Obwohl die intensivmedizinische Patientenversorgung immer besser wird und die meisten Patienten die ITS verlassen können, müssen ca. 10 % nach der Entlassung von der ITS wieder auf die Station aufgenommen werden [16]. Das in dieser Studie gewählte Forschungsdesign ermöglicht neben einer möglichen Identifizierung von Gebrechlichkeit als Risikofaktor für eine Wiederaufnahme auf die ITS, ein tieferes Verständnis für mögliche Einflussfaktoren zu erlangen. Gebrechlichkeit führt zu einer Reduktion der physiologischen Reserven und beeinträchtigt die Fähigkeit des Körpers, akute Belastungen wie Infektionen oder chirurgische Eingriffe zu kompensieren. Dies erhöht die Wahrscheinlichkeit von Komplikationen, z. B. eines verlängerten Krankenhausaufenthalts oder Delirs [17], und erhöht somit das Risiko für Wiederaufnahmen auf die ITS [42, 43].

Ruiz de Gopegui Miguelena et al. (2021; [42]) berichteten in ihrer prospektiven Kohortenstudie mit insgesamt 90 Patienten über eine Wiederaufnahmerate von 28,4 %. Innerhalb der Wiederaufnahmen auf der ITS konnten Ruiz de Gopegui Miguelena et al. (2021) einen Zusammenhang zwischen der Höhe des Gebrechlichkeitsgrads (gemessen mittels CFS) und einer Wiederaufnahme auf die ITS nachweisen (*p* = 0,004).

Sanchez et al. (2020; [43]) zeigten in ihrer prospektiven Kohortenstudie mit insgesamt 977 Patienten ähnliche Ergebnisse. Hier betrug die Wiederaufnahmerate auf die ITS 5 %. In der Gruppe der Wiederaufnahmen konnte ein Zusammenhang zwischen Gebrechlichkeit und einer Wiederaufnahme aufgezeigt werden (*p* = 0,017).

ITS-Wiederaufnahmen erhöhen das Risiko schwerwiegender Komplikationen und sind mit einer erhöhten Mortalitätsrate verbunden [20, 41]. Ein Risikofaktor für die Wiederaufnahme auf die ITS scheint das Alter zu sein [16].

Hinzu kommt, dass die vulnerable Gruppe der älteren, gebrechlichen Patienten auf den Intensivstationen stetig zunimmt [10, 36]. Die Studie von Rosa et al. (2020; [41]) zeigte, dass ITS-Wiederaufnahmen bei gebrechlichen Patienten ein Risikofaktor sowohl für frühe als auch für späte Mortalität sind. Dies unterstreicht die Notwendigkeit, präventive Maßnahmen und engmaschige Überwachung für diese Patientengruppe zu etablieren. Bislang fehlen Untersuchungen, die den Zusammenhang zwischen Gebrechlichkeit und Wiederaufnahmen unter Berücksichtigung der Versorgungsstrukturen in Deutschland analysieren. Diese Forschungslücke ist besonders relevant, da Unterschiede in den Gesundheitssystemen und Ressourcenverfügbarkeiten die Übertragbarkeit internationaler Erkenntnisse einschränken.

Im Gegensatz zu bisherigen Studien strebt diese Studie nicht nur an, Gebrechlichkeit als Risikofaktor für ITS-Wiederaufnahmen zu charakterisieren, sondern auch, vertiefende Erkenntnisse über spezifische Versorgungsmaßnahmen zu gewinnen. Die Ergebnisse dieser Forschung können zum wissenschaftlichen Diskurs über Versorgungskonzepte für gebrechliche Patienten und zum Umgang mit intensivmedizinischen Ressourcen beitragen und stellen eine Grundlage für weitere Forschung dar.

## Limitation

Die vorliegende Studie hat Limitationen. Aufgrund der monozentrischen Durchführung spiegeln die Ergebnisse möglicherweise nur die Besonderheiten des ausgewählten Standorts wider und sind daher nur bedingt generalisierbar. Jedoch können die Ergebnisse der Studie an einem ausgewählten Standort bedeutend für die klinische Praxis sein, indem Optimierungen von Behandlungspfaden aufgezeigt werden könnten.

Eine weitere mögliche Limitation unserer Studie ist, dass ggf. nicht alle potenziell einschlussfähigen Patienten identifiziert werden können. Dies hängt unter anderem von der Methodik und der Durchführung der Patientenaufklärung ab. Zudem wurde die Berechnung der Stichprobengröße auf Basis einer australischen Studie durchgeführt. Die dortigen Versorgungskonzepte könnten von den in Deutschland üblichen Ansätzen abweichen, was potenziell die Wiederaufnahmerate und damit die Übertragbarkeit der Ergebnisse beeinflussen könnte. Zusätzlich ist zu beachten, dass bei der Analyse infektiologischer Komplikationen ausschließlich Pneumonien untersucht werden. Andere Infektionen, wie z. B. katheterassoziierte Infektionen, werden nicht berücksichtigt. Dies kann die Vollständigkeit der Analyse einschränken, da diese ebenfalls eine potenzielle Rolle bei ITS-Wiederaufnahmen spielen könnten. Trotz der Einhaltung und Umsetzung relevanter Gütekriterien im gesamten Forschungsprozess und in den einzelnen Forschungsphasen können insbesondere in der deduktiv-induktiven Dokumentenanalyse die Wahrnehmungen und Interpretationen der Forschenden die Analyse beeinflussen.

Die Forschenden sind während des gesamten Forschungsprozesses um eine lückenlose Datenerhebung bemüht. Jedoch kann nicht ausgeschlossen werden, dass in der Dokumentation durch die Behandlungsteams in der klinischen Praxis Dokumentationslücken entstehen. Dokumentationslücken in den Patientenakten können seitens der Forschenden nicht nachträglich behoben werden. Dies kann die Tiefe und Aussagekraft der qualitativen Dokumentenanalyse einschränken und muss in der abschließenden Diskussion beachtet werden.

## Supplementary Information

Empfehlungen für die Berichterstattung über Studienprotokolle für Observationsstudien und qualitative Studien (ObsQual-Checkliste)

## Data Availability

Die in dieser Studie erhobenen Datensätze können auf begründete Anfrage beim Korrespondenzautor angefordert werden.

## Abkürzungen

ADL: Aktivitäten des täglichen Lebens
CCI: Charlson-Komorbiditäts-Index
CFS: Clinical Frailty Scale
ICDSC: Intensive Care Delirium Checklist
ICU: „Intensive care unit“
IMC: Intermediate-Care-Stationen
ITS: Intensivstation
KISS: Krankenhaus Infektions Surveillance System
MMD: Mixed-Methods-Design
RöThx: Röntgenaufnahme des Thorax
SOFA: Sequential Organ Failure Assessment Score
VAP: Ventilatorassoziierte Pneumonie
