# Supplementary material for: The geriatric syndrome frailty in the context of readmission to the intensive care unit: Study protocol of a mixed-methods study
Source: Med Klin Intensivmed Notfmed. 2025 Apr 23;121(4):293–301. [Article in German] doi: 10.1007/s00063-025-01280-x (PMC13133210; doi:10.1007/s00063-025-01280-x)
Supplement: Supplementary file 1 — Empfehlungen für die Berichterstattung über Studienprotokolle für Observationsstudien und qualitative Studien (ObsQual-Checkliste) [file 63_2025_1280_MOESM1_ESM.pdf]

Development and validation of observational and qualitative study protocol reporting checklists for novice researchers (ObsQual checklist)

Observational study protocol checklist (Cross-sectional, Case-control and cohort study design) with educational elements

Item	Item No.	Explanation/Recommendation	Page No.
Title	1	Indicate the study's design with a commonly used term in the title. (if study design not mentioned but title is informative enough to determine the study design)	Titel Page
Introduction/ Background/rationale	2	Explain the scientific background and rationale for the investigation being reported.<br>This section requires references from current literature.	6-7
	3	The study is necessary in comparison with current evidence.<br>It is important to state the importance of this study in the absence of evidence. If evidence is already available to address the study objectives, consider other objectives of study that addresses the knowledge gap.	6-7
Objectives	4	State specific objectives, including any prespecified hypotheses<br>An objective should be SMART (Specific, Measurable, Achievable, Realistic, Time-bound)	7, 8
Methods Study design	5	Present key elements of study design early in the paper.<br>A one paragraph of overview of the study design.	8
Setting	6	Describe the setting, locations, and relevant dates, including periods of recruitment, exposure, follow-up, and data collection	9-10
Participants	7	*Cohort study*—Give the eligibility criteria, and the sources and methods of selection of participants. Describe methods of follow-up<br><br>Eligibility/selection criteria are designed to address inclusion or exclusion of participants with certain characteristics (factors) that may influence the analysis. It is a method used to control confounding factors.<br>E.g. if a factor such as young age or a gender can affect the outcome, specifically inclusion or exclusion can control the confounding factors.<br><br>Method of follow-up to be described. Mention the time of follow-up (monthly, yearly, etc). The personnel that conduct the follow-up and materials used to record the data.<br>The same description is also applicable for database analysis. Hence, understanding of how the data was collected in the database is important for the description.	10-12
Variables	8	Clearly define all outcomes, exposures, predictors, potential confounders, and effect modifiers. Give diagnostic criteria, if applicable	11,12

Data sources/ measurement	10	For each variable of interest, give sources of data and details of methods of assessment (measurement). Describe comparability of assessment methods if there is more than one group<br>This section may be combined with above Item 12 when defining the variable.	11,12
Bias	11	Describe any efforts to address potential sources of bias Provide rationale of certain methods used to address potential bias.<br>E.g., Stratified random sampling was used to prevent imbalance of gender being sampled.	11,12
Study Size	12	Explain how the study size was arrived at<br>Sample size is usually calculated and to be stated. If it is not, there should be a clear indication of why the sampling is not required such that all participants in the dataset will be included.<br>For a pilot/feasibility study, they are meant to assess feasibility of certain aspect of the study before conducting a full study (where no literature that can be used to calculate sample size).	12-13
Quantitative variables	13	Explain how quantitative variables will be handled in the analyses. If applicable, describe which groupings will be chosen and why	11,12
Statistical methods	14	Describe all statistical methods, including those used to control for confounding.<br><br>The methods such as T-test, Chi-square, regression, etc must be stated.<br>To control for confounding factors in statistics, a multivariable model is often used i.e. multiple linear/ logistic regression, Cox proportional hazard.	13-14
	14	Describe any methods used to examine subgroups and interactions.	13,14
	15	Explain how missing data will be addressed<br>Missing data is almost always present, and it should be planned on how to deal with. E.g. exclude missing data if less than 20 %. Intent-to-treat by “worst-case best-case scenario”, multiple imputation, etc.	13,14
	16	*Cohort study*—If applicable, explain how loss to follow-up will be addressed<br>This can be addressed by design and by statistics. By design, it should be written in the Item 10 (bias).	13,14
Appendices Biological<br><br>specimens		Plans for collection, laboratory evaluation, and storage of biological specimens for genetic or molecular analysis in the current trial and for future use in ancillary studies, if applicable	Not applicable

Qualitative study protocol checklist with educational elements

Item	Item No.	Explanation/Recommendation	Page No.
Title	1	Indicate the study's design with a commonly used term in the title. (if study design not mentioned but title is informative enough to determine the study design)	Titel Page
Introduction/ Background/rationale	2	The study is necessary in comparison with current evidence.<br><br>It is important to state the importance of this study in the absence of evidence. If evidence is already available to address the study objectives, consider other objectives of study that addresses the knowledge gap.	6-7
Aim/Objectives	3	State specific objectives, including any prespecified hypotheses<br>An objective should be SMART (Specific, Measurable, Achievable, Realistic, Time-bound)	7,8
Methodological orientation and Theory	4	What is the methodological orientation that underpins the study? e.g. grounded theory, discourse analysis, ethnography, phenomenology, content analysis	8
Participant selection<br><br>Sampling	5	How will participants be selected? e.g. purposive, convenience, consecutive, snowball	10
Description of sample	6	What are the important characteristics of the sample? Who are you targeting for inclusion in the study and why?	10
Method of approach	7	How will study participants be approached?	11, 16
Sample size	8	How many participants are anticipated being included in the study?	12-13
Setting of data collection	9	Where will the data be collected?	9, 10
Presence of non<br><br>participants	10	Who will be present during the data collection activities? Anyone besides the participants and researchers?	Not applicable
Data recording	11	How will the qualitative data be recorded?	14
Field notes	12	Will field notes be made? Will they be included for data analysis purposes?	Not applicable
Duration	13	What is the anticipated duration of the interviews or focus group discussions?	Not applicable
Qualitative analysis	14	Types of analysis that can be used: content analysis, thematic analysis, narrative analysis, discourse analysis, etc.	15

Member checking	15	Do you anticipate returning data or results to participants for accuracy and/or validation purposes? If none, please state as NA.	Not applicable

Quelle: Low GK, Subedi S, Omosumwen OF et al. (2024) Development and validation of observational and qualitative study protocol reporting checklists for novice researchers (ObsQual checklist). Eval Program Plann 106:102468
